# Molecular characterization of the *netrin-1 UNC-5* receptor in *Lucilia sericata* larvae

**DOI:** 10.3934/genet.2019.3.46

**Published:** 2019-08-08

**Authors:** Tahereh Karamzadeh, Hamzeh Alipour, Marziae Shahriari-Namadi, Abbasali Raz, Kourosh Azizi, Masoumeh Bagheri, Mohammad D. Moemenbellah-Fard

**Affiliations:** 1 Research Center for Health Sciences, Institute of Health, Shiraz University of Medical Sciences, Shiraz, Iran; 2 Department of Medical Entomology, School of Health, Shiraz University of Medical Sciences, Shiraz, Iran; 3 Malaria and Vector Research Group, Biotechnology Research Center, Pasteur Institute of Iran, Tehran, Iran

**Keywords:** molecular profile, *Netrin-1 UNC-5 receptor*, *Lucilia sericata*, blowfly larvae

## Abstract

Larval therapy with *Lucilia sericata* is a promising strategy in wound healing. Axon guidance molecules play vital roles during the development of the nervous system and also regulate the capacity of neuronal restoration in wound healing. *Netrin-1*, one of the proteins that larvae secrete, plays a useful role in cell migration and nerve tissue regeneration. The *UNC-5 receptor* combines with a *netrin-1* signal and transmits the signal from one side of the membrane to the other side, initiating a change in cell activity. In the current study, we identified the full length of the *UNC-5 receptor* mRNA in *L. sericata* using different sets of primers, including exon junction and specific region primers. The coding sequence (CDS) of the *UNC-5 receptor* was sequenced and identified to include 633 base-pair nucleic acids, and BLAST analysis on its nucleotide sequence revealed 96% identity with the *Lucilia cuprina* netrin-1 *UNC-5 receptor*. The protein residue included 210 amino acids (aa) and coded for a protein with 24 kD weight. This gene lacked the signal peptide. Furthermore, the UPA domain is conserved in *UNC-5*. It lied at the interval of 26–131 aa. We identified the CDS of *netrin-1* *UNC-5 receptor* in *L. sericata*. It could be applied to research activities implementing a new essential component design in wound healing.

## 1. Introduction

The use of *Lucilia sericata* (*L. sericata*) blowfly larvae in diabetic wound healing have previously been reported [1]. One of the enzymatic groups with an essential role in wound injury is the family of netrin-1 molecules [2]. One of the receptors in this group is the *UNC-5 receptor*. The *UNC-5* family of receptors mediate the repellent response to *netrin-1*. Netrins are a group of extracellular proteins that are present in invertebrates, nematodes, and insects [3],[4]. They play certain roles in axon guidance and development of the nervous system. They transmit their activity via two different receptors. The role of *UNC-5* is in axon repelence, while the molecular function of the other receptor ‘Deleted in Colorectal Cancer’ (DCC) is to attract and repel axons by *netrin-1* [5]. In addition, other studies showed that netrin-1 has other receptors such as the adenosine 2B receptor (A2BAR) [6]. According to previous studies, *netrin-1* mediated through different receptors may regulate diverse signaling pathways and play distinct roles in physiological and pathophysiological conditions [7],[8]. Netrin-1 has recently attracted increasing interests in wound repair progression in diabetic complications [9]. In *Drosophila melanogaster* fruitfly, netrin A and netrin B have been identified [10]. Both netrins A and B are expressed by the midline cells during the initial period of formation in the ventral nerve cord [10],[11]. They are involved in many functions such as axon guidance, control survival or apoptosis, and tumor suppression by their receptors [12],[13]. Netrins have a variety of receptors; the central receptors are *DCC* and *UNC-5A-D* in human and *UNC-5H1-4* in rodents [14]. *DCC receptors* were identified as the first netrin receptors [15]. The UNC-5 is one of the netrin-1 receptors usually expressed in embryonic and adult mammals. The *UNC -5 receptors* can control apoptosis in the presence or absence of *netrin-1* protein. Transplantation of bone marrow mesenchymal stem cells that produce *netrin-1* improved the function of the sciatic nerve after injury. This method may be used in the future in the treatment of nerve injury [16]. Netrin-1, a protein recognized in the guidance of commissural axons, plays a similar function in angiogenesis. Furthermore, it was shown that the *netrin-1 UNC-5* receptor is expressed in capillaries. More studies on this new group of molecules will be interesting. *Netrin-1*, an enzyme known in the guidance of commissural axons, acts similarly in angiogenesis. It was also shown that one of the *netrin-1* receptors is expressed at the apical cells of growing small blood vessel. It would be interesting to survey this new group of proteins. in the future. Netrins and netrin receptors have important roles with respect to angiogenesis in wound healing [17]. Some studies showed that netrin-1 also stimulates angiogenesis *in vivo* and enhances the response to vascular endothelial growth factor [18]. Whereas one of the methods currently considered by the practitioners in the treatment of wounds and approved by the FDA in 2004 is the maggot therapy method. It also occurs in the process of repairing the nerve tissue in wound healing [19]. *Netrin-1*, as a macrophage maintenance signal in fat tissue during obesity, boosts chronic inflammation and insulin resistance*. Netrin-1*, as a significant signal, reconciles the dynamic crosstalk between inflammation and constant erosion of the extracellular matrix in abdominal aortic aneurysms. *Netrin-1* generates a steady intracellular calcium flow necessary for the transcriptional regulation and persistent catalytic activation of matrix metalloproteinase-3(*MMP3*) by vascular smooth muscle cells [20],[21]. The *netrin-1* protein with the *UNC-5* and *DCC receptors* are shown to be implicated in axonal regeneration after injury [22].

This study was conducted to identify and characterize the *netrin-1* UNC-5 receptor in larvae of *L. sericata*. This blowfly is a species in the family Calliphoridae, Class Insecta with a critical role in maggot therapy [23]. Since the *L. sericata* larvae are used in maggot therapy and involved in the repair of the damaged nerve tissue, therefore, the identification of the nucleic acid sequence of the *UNC-5* receptor of *L.sericata* and its recombinant expression in the future can be effective in producing drugs for the treatment of ulcers. In this way, using the recombinant protein, it also increases the *netrin-1* secretion and accelerates the recovery of the damaged nerve tissue.

## 2. Materials and methods

### 2.1. Rearing of L. sericata larvae

Experiments conducted on the second instar larvae of *L. sericata* from a colony that had been reared at the School of Health insectarium, Shiraz University of Medical Sciences (SUMS) under constant conditions. Adults were exposed to a 12 hours L/D cycle at a relative humidity of 40–50% and temperature range of 18–25 °C. The larvae were fed on beef. Accurate species identification was routinely confirmed using valid pictorial taxonomic key based on morphological characterization.

### 2.2. Primer design

**Figure 1.**
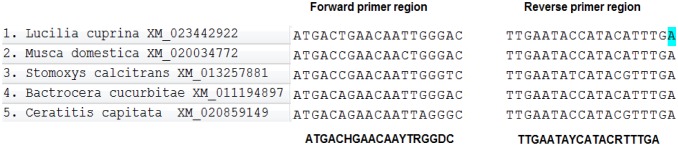
The alignments of five *UNC5-netrin* sequences gene by MEGA 0.6 software. The forward (NetF1) and reverse (NetR650) primer sequences are shown at the bottom (Y = C/T, R = A/G, H = A/T/C, D = A/G, R=A/G). The sequences used here are available from the GenBank under the following accession numbers: *Ceratitis capitata* (XM_020859149), *Bactrocera cucurbitae* (XM_011194897), *Musca domestica* (XM_020034772), *Stomoxys calcitrans* (XM_013257881), and *Lucilia cuprina* (XM_023442922).

Since the *L. sericata* genome has not been sequenced yet and based on our previous study [24], we decided to design a gene-specific primer for the identification of netrin-1 UNC-5 receptor [24]–[28]. First, the mRNA sequences of netrin-1 from different species of Diptera such as *Ceratitis capitata* (XM_020859149), *Bactrocera cucurbitae* (XM_011194897), *Musca domestica* (XM_020034772), *Stomoxys calcitrans* (XM_013257881), and *Lucilia cuprina* (XM_023442922) were obtained from NCBI and aligned using the MEGA 6 software. Then, degeneration primers were determined based on conserve regions (Figure 1). After analysis, three regions were chosen to design gene-specific primers (GSPs). Three exon junction primers: NetF1 (5′-AATATATTGGAGTTTATCGG-3′), NetF2 (5′- ATATATTGTCGAATAATTC-3′), and NetR650 (5′- CGATATACACGAGGCAGTAG -3′) were designed to determine the full length of the target gene as forward and reverse primers, respectively. The normal size bound was 633 and 290 bp, respectively. Primers were designed by Gene Runner 0.4, Oligo 0.7 and BLAST (online tool) softwares.

### 2.3. RNA extraction

Total RNA was extracted from the salivary glands of the second instar larvae using high pure RNA isolation kit (Roche Company, Germany) and the extracted RNAs were treated by DNaseI (Roche, Germany), both according to the manufacturer's instruction and then stored in −70 °C.

### 2.4. cDNA synthesis

Extracted RNA was used for the first strand cDNA synthesis. Then reverse transcription (RT) reaction was performed according to RevertAid First Strand cDNA Syn. kit (Fermentas Company) by the random hexamers primer.

### 2.5. Polymerase chain reaction (PCR)

All polymerase chain reactions (PCRs) were performed in a 20 µL total volume for 35 cycles using 2 µL of synthesized cDNA or 150 ng genomic DNA in each cycle as a template. The reaction mixture contained 400 nM of each primer, 1.5 mM MgCl_2_, 1 unit *Taq* DNA polymerase, 0.2 mM dNTPs, 2 µL 10 × reaction buffer, and the final volume was adjusted to 20 µL with double distilled water (dd H_2_O). The amplification program was set as follows: 5 min at 94 °C; followed by 35 cycles of denaturation at 94 °C for 30 s, annealing at 58 °C for 30 s, extension at 72 °C for 30 s; and an additional final extension at 72 °C for 10 min. The amplified amplicons were purified using the DNA gel purification kit (GF-1 Vivantis, Malaysia).

### 2.6. Sequencing

Expected size bonds after gel purification were sequenced, and their analysis was performed by Chromas (Version 2.31, 2005), DNA Star (Version 7.10, 2006), MEGA6 (Build 5110426, 2011) and BLAST by NCBI online. Amplicons with a size close to the predicted range sequenced using GSPs forward and reverse primers.

### 2.7. Bioinformatics

All primers were designed using the Gene Runner (version 0.4) and Oligo 0.7 software. Alignments were performed by the MEGA software (version 6.0), and their specificity for PCR was checked by nucleotide BLAST on NCBI (http://blast.ncbi.nlm.nih.gov/Blast.cgi).

## 3. Results

This study identified 633 base-pair nucleic acids (mRNA) of the netrin-1 UNC-5 receptor. The expected protein was determined by *in silico*. The protein residue included 210 amino acids and coded a protein with 24 kD weight (Figure 4). Besides, the residue of amino acids lacked the signal peptide. The UPA domain was conserved in *UNC-5*. It was situated at the interval of 26–131 aa. We identified the CDS of *netrin-1* *UNC-5* receptor in *L. sericata* which could be involved in research activities applied in the design of a new essential component in diabetic wound healing.

**Figure 2.**
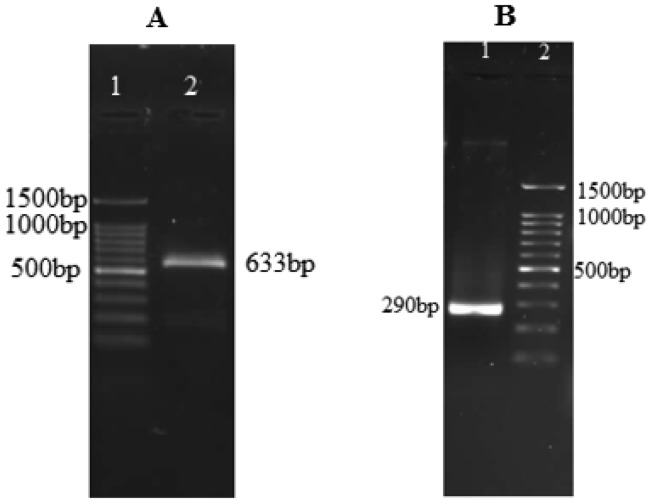
(A) 1: Marker 100bp, 2: UNC-5 netrin-1 receptor in *Lucilia sericata* by primer of NetF1 and NetR650 (633bp), (B) 1: *UNC5* *netrin-1* receptor in *Lucilia sericata* by primer of NetF2 and NetR650, 2: Marker 100bp (290bp).

**Figure 3.**
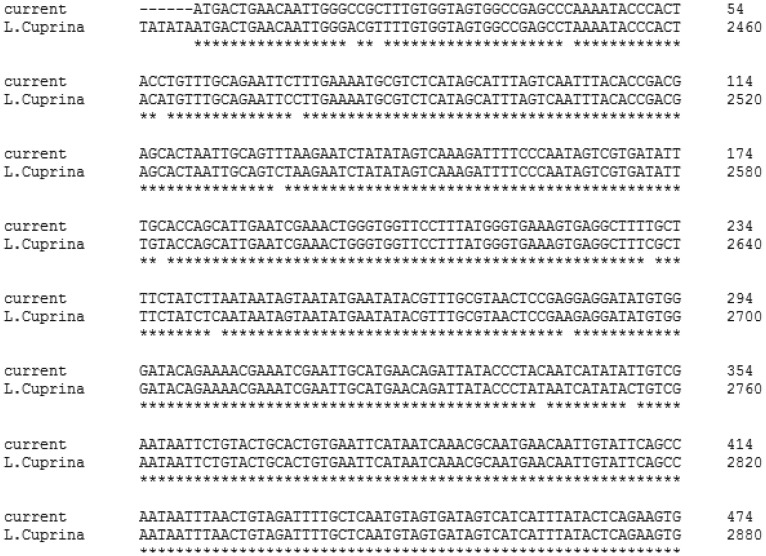
Comparison of *L. sericata* *netrin-1* receptor *UNC-5* mRNA sequence to *L. cuprina* by Clustal Omega software online.

In the reactions performed, the coding sequence of the *netrin-1 UNC-5* receptor from the salivary gland of *L. sericata* was determined. The PCR reactions were carried out using a combination of NetF1, NetF2, and NetR633 primers, which resulted in finding exactly two amplicons with the expected sizes of 633 bp and 290 bp (Figure 2). Sequencing 633bp fragment and BLAST analysis on its nucleotide sequence revealed 96% identity with the *Lucilia cuprina* *netrin-1* receptor *UNC-5* mRNA sequence (Figure 3). This part of the gene sequence was submitted to GenBank (GenBank: MG009433 and MG009434). This study showed that the protein residue did not have a signal peptide.

**Figure 4.**
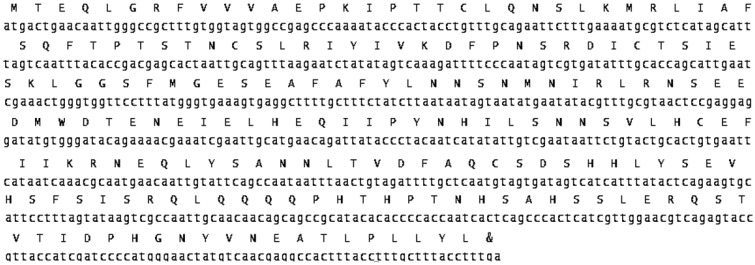
Amino acid and nucleic acid sequences of *L. sericata UNC-5 receptor.*

**Figure 5.**
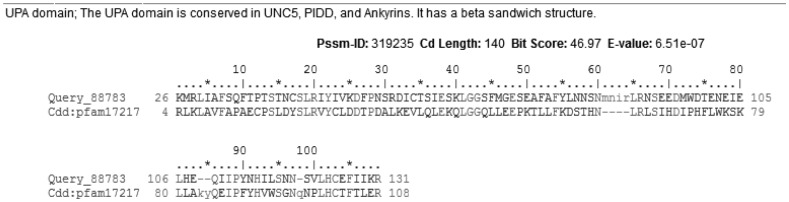
The conserved domain of *L. sericata* netrin-1 UNC-5 receptor evaluated by NCBI online.

Moreover, the conserved domain of *L. sericata* *netrin-1 UNC-5* receptor was calculated by NCBI. The UPA domain was conserved in UNC-5, PIDD, and Ankyrins. It had a beta sandwich structure, interval of 26–131 aa, and E-value = 6.51e-07 (Figure 5).

**Figure 6.**
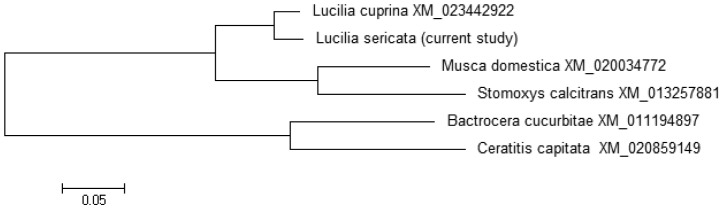
The phylogenetic tree of *L. sericata* sequence compared to five other gene sequences named in the illustration and constructed by the MEGA 0.6 software.

Following determination of nucleic acid sequence, the phylogenetic tree was constructed using MEGA6 software based on Neighbor Joining method with bootstrapping to provide confidence for tree topology.. According to the phylogenetic tree of L*. sericata*, nucleotide sequences of the submitted *netrin-1 UNC-5* receptor sequences in the *Musca domestica*, *Stomoxys calcitrans*, *Bactrocera*, and *Ceratitis capitata* gene sequences, as well as *L. sericata* and *L. cuprina* were grouped in a cluster different from other mentioned genes (Figure 6).

## 4. Conclusions

We identified the developmental sequence of the *netrin-1 UNC-5* receptor in *L. sericata* for the first time. Total mRNA was identified as 633 bp. The CDS of netrin-1 receptor in *L. sericata* was identified with 633 bp nucleic acids and this receptor can code a protein with 24 kDa weigth. *Netrin-1* is one of the enzymes secreted from the salivary glands of *L. sericata*, which are laid on wounds or injuries in the treatment by the method of maggot therapy.

The discovery of netrins constitutes a grand leap onto the evolution of developmental control mechanisms among neuronal cells. The leading role of netrin-1 and netrin-1 receptors helps to repair injured nerve tissues. Within insects, divergent sets of axon guidance molecules, such as the midline repellant Slit and its *Roundabout* (Robo) receptors, are usually implicated to achieve equal developmental outcomes [29]. One likely mechanism leading to precise control of axonal guidance is found to be the duplication through use of orthologs and functional diversification of similar pathway components.

Some studies on the identification of enzymes secreted from *L. sericata* such as the study of Telford in 2010 on chymotrypsin and Cerovsky in 2010 on lucifensin have been published [30],[31]. All cells used in the inflammation such as neutrophils, monocytes, macrophages, synovial fibroblasts, and bone destruction characterize this model of arthritis express the *UNC-5B* and *netrin-1* in addition to its effects on leukocyte migration [32],[33]. *Netrin-1* regulates the inflammatory response of neutrophils and macrophages via suppression of cyclooxygenase 2–mediated prostaglandin E2 production during ischemic acute kidney injury [34],[35]. Netrin-1, acting via the *Unc5B* receptor, induces activation of peroxisome proliferator-activated receptor-γ and other signaling pathways, which causes suppression of IκB degradation and inactivation of NF-κB transcription factor [34]. *Netrin-1* contributes to the regulation of leukocyte migration and inflammation in peripheral tissues [36] to reduce local wound and inflammatory responses [37] and to stimulate resolution actions and production of resolving [34]. In contrast to these generally anti-inflammatory activities, netrin-1 performs a role in the collection of macrophages at inflamed sites of skin and tissue, like atherosclerotic plaque [38]. During ischemia–reinventing blood flow, netrin-1 enzyme is significantly down-regulated, *UNC-5B* mRNA expression is increased, and the *DCC* receptor is not changed significantly [34].

All these and other studies point to the fact that certain enzymes or other molecules derived from larval blowflies or alternative insects could be conducive to the repair, regeneration, and swift resolution of impaired human body tissues. They are thus a promising field of research activity.

One of the drawbacks in the current study was that a shortage of basic raw materials essential to launch a wider scale study was impeding to assist the resolution of all research inquiries in this field of study.

It is concluded that the coding sequence of the *netrin-1 UNC5* receptor in *L. sericata* larvae resembles those of other insect species. The findings presented in this research article could be manipulated in future endevours to enhance wound healing using maggot therapy.
